# Osteohistology and growth dynamics of the Brazilian noasaurid *Vespersaurus paranaensis* Langer et al., 2019 (Theropoda: Abelisauroidea)

**DOI:** 10.7717/peerj.9771

**Published:** 2020-09-15

**Authors:** Geovane Alves de Souza, Marina Bento Soares, Arthur Souza Brum, Maria Zucolotto, Juliana M. Sayão, Luiz Carlos Weinschütz, Alexander W.A. Kellner

**Affiliations:** 1 Programa de Pós-Graduação em Zoologia (PPGZoo), Museu Nacional, Universidade Federal do Rio de Janeiro, Rio de Janeiro, Rio de Janeiro, Brazil; 2 Laboratory of Systematics and Taphonomy of Fossil Vertebrates, Departamento de Geologia e Paleontologia, Museu Nacional, Universidade Federal do Rio de Janeiro, Rio de Janeiro, Rio de Janeiro, Brazil; 3 Departamento de Geologia e Paleontologia, Museu Nacional, Universidade Federal do Rio de Janeiro, Rio de Janeiro, Rio de Janeiro, Brazil; 4 Núcleo de Biologia, Centro Acadêmico de Vitória, Universidade Federal de Pernambuco, Vitória de Santo Antão, Pernambuco, Brazil; 5 Centro Paleontológico, Universidade do Contestado, Mafra, Santa Catarina, Brazil

**Keywords:** Dinosauria, Cretaceous, Vespersaurus, Brazil, Abelisauroidea

## Abstract

Although the knowledge of bone histology of non-avian theropods has advanced considerably in recent decades, data about the bone tissue patterns, growth dynamics and ontogeny of some taxa such as abelisauroids are still limited. Here we describe the bone microstructure and growth dynamics of the Brazilian noasaurine *Vespersaurus paranaensis* using five femora and six tibiae and quantify the annual growth marks through retrocalculation of missing ones to estimate ontogenetic ages. The femoral series comprises four femoral histological classes (FHC I-IV), varying from two annuli or LAGs to seven LAGs. Femora show that sexual maturity was achieved around the seventh to tenth year of life, whereas the tibiae suggest it was earlier (around three to five years old). Tibiae represent three histological classes (THC I-III) displaying from three to nine LAGs. Two tibiae (THC III) exhibit an external fundamental system indicating that these specimens reached full skeletal size. The heterogeneous maturity observed in *Vespersaurus* hind limb bones could result from diﬀerential allometry scaling between femora and tibiae length with the body length. The predominant parallel-fibered bone matrix suggests that *Vespersaurus* grew more slowly than most theropods, including other abelisauroids, in a pattern shared with the noasaurines *Masiakasaurus knopfleri* from Madagascar and CPPLIP 1490 from Brazil. This deviation from the typical theropod growth pattern may be mainly correlated with small body size, but also may related to resource limitation imposed by the arid climate prevailing in southwestern Gondwana during Cretaceous. Moreover, given the ecological and phylogenetic similarities among these taxa, such features would probably be apomorphic within Noasauridae.

## Introduction

In recent years, studies on bone histology of dinosaurs in general and non-avian theropods in particular have provided new insights about growth and developmental strategies in relatively well-sampled taxa such as coelophysoids (Chinsamy, 1990), *Tyrannosaurus rex* (Horner & Padian, 2004; Padian, Horner & Ricqlès, 2004; Lee & Werning, 2008) and *Allosaurus agilis* (Bybee, Lee & Lamm, 2006). In contrast, many dinosaur groups remain undersampled, with no well-known histological framework, especially those based on more incomplete taxa (Veiga et al., 2019). Among theropod dinosaurs, the Abelisauroidea is one such undersampled group. This lack of information means that the bone microstructure of abelisauroids is not well understood, preventing inferences regarding ecology and physiology, and comparison with other theropod dinosaurs, as well as preventing the placement of their growth patterns into a broader evolutionary context.

Abelisauroidea sensu Wilson et al. (2003) comprises the main predator dinosaurs of Gondwana predators during the early Late Cretaceous, displaying one of the greatest degrees of taxonomic and ecologic diversity among non-avian theropod lineages (Carrano & Sampson, 2008; Novas et al., 2013; Tortosa et al., 2014, Brusatte, Candeiro & Simbras, 2017; Delcourt, 2018). Despite such diversity, only a few taxa belonging to two of the largest clades within Abelisauroidea, Abelisauridae and Noasauridae, have been the subject of paleohistological studies.

Histological sections performed in the Late Cretaceous abelisaurids *Aucasaurus garridoi* (ca. 6.2 m body length) and *Quilmesaurus curriei* (ca. 5.3 body length) revealed a fibrolamellar tissue, with intensive remodeling in the cortex (Cerda, 2015; Baiano & Cerda, 2017), typical of fast-growing animals (Francillon-Vieillot et al., 1991; Padian, De Riclès & Horner, 2001; De Margerie, Cubo & Castanet, 2002). Completing the list of paleohistologically examined abelisaurids is the unnamed specimen MMCh-PV 69, smaller (ca. 4.2 m body length) than *Aucasaurus* and *Quilmesaurus*, despite being somatically mature. The femur of MMCh-PV 69 exhibits more organized intrinsic fibers and a lesser degree of bone remodeling, in the femur, which would indicate a relatively lower growth rate, despite the presence of woven bone in the ribs (Canale et al., 2016).

**Figure 1:**
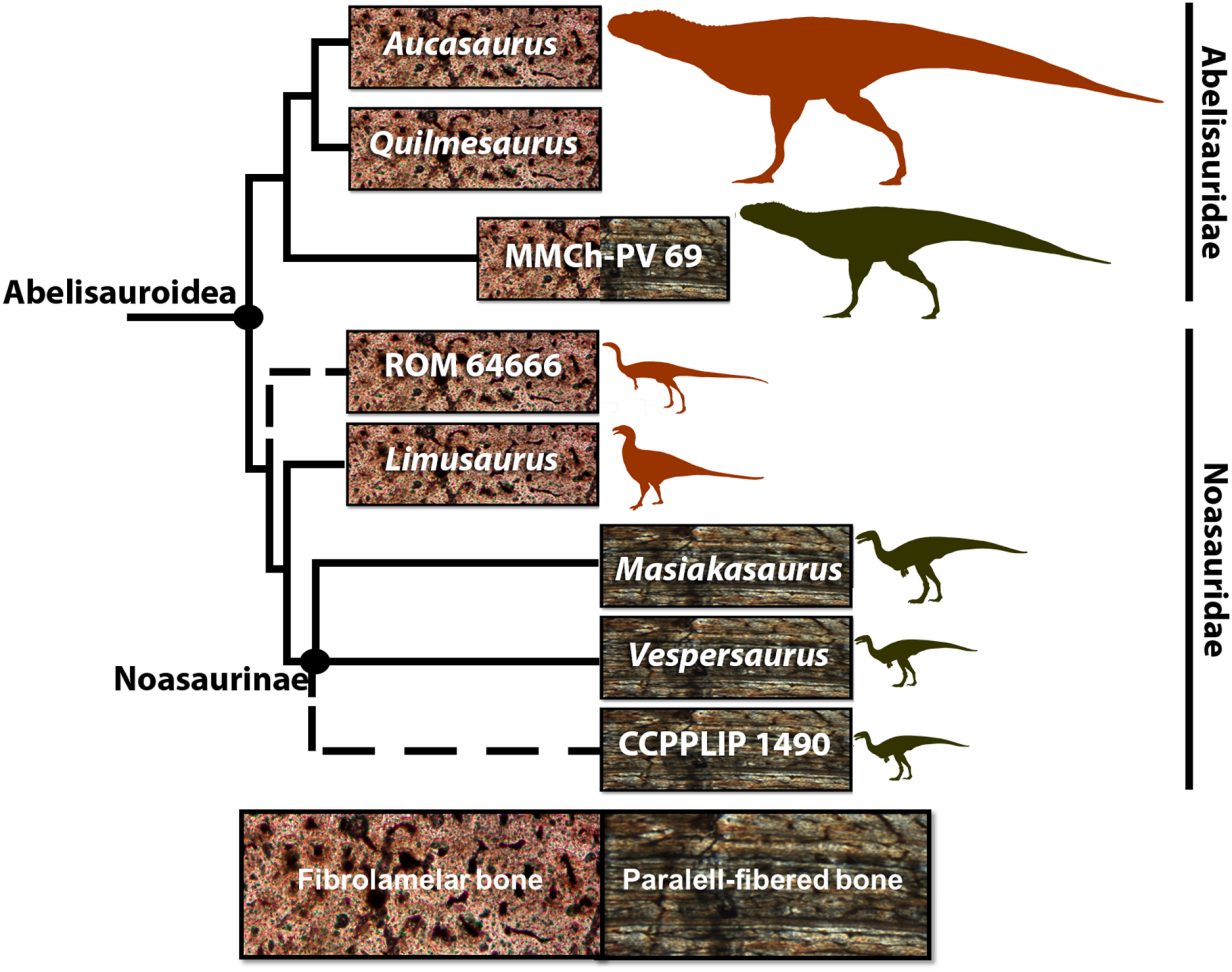
Cladogram of Abelisauroidea, comparing body length and primary bone tissue type of the histologically analyzed *taxa*. Sizes in scale based on BL estimations of Grillo & Delcourt (2017). From the larger to the smaller: *Aucasaurus garridoi* (6.2 m), *Quilmesaurus curriei* (5.3 m), MMCh-PV 69 (4.2 m), ROM 64666 (2 m), *Limusaurus inexplicabilis* (1.7 m), *Masiakasaurus knopfleri* (1.8 m), *Vespersaurus paranaensis* (1.5 m) and CCLPP 1490 (1.4 m). Phylogenetic hypothesis based on Rauhut & Carrano (2016) and Langer et al. (2019). Branch lengths indicate distance from the plesiomorphic fibrolamellar pattern.

Noasauridae comprises small-sized abelisauroids with procumbent teeth (at least in some species), long necks, larger arms and longer skulls compared to their sister taxon, Abelisauridae (Xu et al., 2009; Carrano, Loewen & Sertich, 2011). Two clades are recovered within Noasauridae: The Middle-Late Jurassic Elaphrosaurinae and the Cretaceous Noasaurinae (Rauhut & Carrano, 2016; Wang et al., 2017; Langer et al., 2019). Some putative noasaurids such as *Deltadromeus agilis* (ca. 5.9 m body length) from the Upper Cretaceous of Kem Kem region of Morocco (Wilson et al., 2003; Sereno et al., 1996; Sereno, Wilson & Conrad, 2004) and *Limusaurus inexplicabilis* (ca. 1.7 m body length) from the Upper Jurassic of China (Xu et al., 2009; Wang et al., 2017) are still of uncertain taxonomic position, being nested as basal Ceratosauria (Ezcurra, Agnolin & Novas, 2010; Pol & Rauhut, 2012) or as Elaphrosaurinae in more recent phylogenies (Rauhut & Carrano, 2016; Wang et al., 2017; Langer et al., 2019) like that shown in Fig. 1. *Limusaurus* and the noasaurine *Masiakasaurus knopfleri* (ca. 1.8 m body length) from the Upper Cretaceous of Madagascar (Carrano, Sampson & Forster, 2002), are the best-preserved noasaurids, and both are represented by several individuals within different size classes (Xu et al., 2009; Lee & O’Connor, 2013; Wang et al., 2017). These specimens have provided an opportunity to explore noasaurid osteohistology, growth dynamics and ontogeny. *Masiakasaurus* is characterized by the predominance of parallel-fibered bone that suggests a slow growth rate even in early ontogenetic stages (Lee & O’Connor, 2013), whereas the growth series of *Limusaurus* revealed fibrolamellar bone, characteristic of fast bone deposition and high growth rates. Two other Late Cretaceous isolated femora, ROM 64666 (a putative noasaurid from the Kem Kem beds, Morocco) and CPPLIP 1490 (a noasaurine from the Late Cretaceous of Bauru Group, Brazil) have already been analyzed histologically. ROM 64666 is a young juvenile, despite its larger size compared to the most noasaurids (204 mm length, equivalent to the largest specimens of *Masiakasaurus*), characterized by a fast-growing fibrolamellar bone complex and lack of growth marks (Evans et al., 2015). In the femur CPPLIP 1490 (preserved length of 132 mm), the compacta is primarily composed of slowly forming parallel-fibered bone tissue (Martinelli et al., 2019).

These noasaurids represent significant histological samplings among theropod taxa. However, as well as for the whole of Abelisauroidea, their paleobiological aspects, such as histovariability (both within a single skeleton and different individuals), longevity, growth strategies, and how these could be evolutionarily related to environmental constraints, remain poorly known.

The type series of the recently described Brazilian Noasaurinae *Vespersaurus paranaensis*, recovered from a Bauru Basin stratum located in Northwestern Paraná state, is composed of cranial elements, vertebrae, left humerus, left radius, a right tibial shaft, tarsals, metatarsals and pedal phalanges, achieving ca. 1.5 m body length (Langer et al., 2019). Apart from the type series (which may be a composite; Kellner et al., 2019), hundreds of bones from the same sandstone beds are housed in the fossil vertebrate collection of the Centro Paleontológico (CENPALEO) of Universidade do Contestado in Santa Catarina state, Brazil (Kellner et al., 2019). This set of bones exhibits high element repetition and differs slightly in size, particularly concerning the tibiae and femora. Unfortunately, not all the available bones have the entire shaft preserved, which limits more comprehensive studies on skeletochronology and comparative ontogenetic growth approaches, but they still provide an opportunity to analyze the bone microstructure of this taxon as well as discussions about growth rates and age estimates.

The purpose of this work is to describe the microstructural bone organization of *Vespersaurus paranaensis*, specifically femora and tibiae, seeking, whenever possible, information on growth rates and ontogenetic stage of the analyzed specimens. This study provides new palaeohistological information, expanding knowledge of abelisauroid biology, adding to the other Cretaceous noasaurids histologically sampled, and allowing discussions about the possible establishment of a shared pattern of bone tissue and growth rate and its relationship with environmental constraints prevalent during the Cretaceous in Gondwana.

### Geological settings

The “Caiuá Group” (Goio Êre, Rio Paraná and Santo Anastácio formations), together with the chronocorrelated unit, the Bauru Group, corresponds to a lithostratigraphic unit within the Cretaceous Bauru Supersequence, located in a central-southern part of the South American platform (Fernandes & Coimbra, 1996; Fernandes et al., 1994; Batezelli & Ladeira, 2016; Menegazzo, Catuneanu & Chang, 2016). The Rio Paraná and Goio Êre formations were deposited under arid conditions characteristic of an inner eolian desert system, being composed of reddish sandstones with medium to large sets (20 m width) cross-bedding stratification and often exhibiting calcareous cementation (Fernandes et al., 1994). There is no consensus about the age of the Caiuá unit, but an Aptian–Albian, Aptian–Cenomanian or even Coniacian–Campanian age have been hypothesized (Fúlfaro & Barcelos, 1993; Fúlfaro et al., 1999; Dias-Brito et al., 2001; Batezelli et al., 2014; Manzig et al., 2014; Kellner et al., 2019).

Fossil vertebrate specimens (pterosaurs and dinosaurs) come from the “Cemitério dos Pterossauros” site, located in the Cruzeiro do Oeste municipality, Northwestern Paraná State, Brazil. The outcrop is located on a rural road in the outskirts of Cruzeiro do Oeste city. The depositional environment is interpreted as a humid inter-dune depositional system within the Caiuá Formation desert system (Kellner et al., 2019). The quarry has three stratigraphic levels where *Vespersaurus* and the pterosaurs *Caiuajara dobruski* and *Keresdracon vilsoni* were recovered (Manzig et al., 2014; Langer et al., 2019; Kellner et al., 2019). The levels are separated by approximately 25 cm from each other. All three levels exhibit mostly disarticulated specimens varying in size. The whole bone assemblage shows little sorting and different weathering stages, but in general, few signs of abrasion are observed.

## Materials and Methods

Specimens are housed in Centro Paleontológico (CENPALEO) of Universidade do Contestado, Mafra city, Santa Catarina State, Brazil. All bones here sampled come from the same site where the type series of *V. paranaensis* was found. Since no other theropod was recovered or identified from this area and based on the overall similar morphology of the specimen studied here with the composite material attributed to this species (see Kellner et al., 2019), we regard our material as belonging to the same taxon.

We sectioned five femora and six tibiae. These appendicular elements were chosen for the following reasons: (1) femora and tibiae were sampled in previous paleohistological studies of Abelisauroidea (Lee & O’Connor, 2013; Martinelli et al., 2019), allowing comparisons; and (2) the hind limb elements recovered from *V. paranaensis* vary slightly in size and presumably reflect different histological classes. The femora circumferences vary from 72% to 78% of the maximum femoral circumference of *Vespersaurus* found so far, whereas the tibiae vary from 91% to 93% of the maximum tibial circumference. Regarding the histological sections, midshafts are the most suitable for accessing the best growth record (Stein & Sander, 2009). However, some *Vespersaurus* elements in this sample consist of only proximal or distal ends. We do not exclude the fragmented elements because the potential they offered to reveal unprecedented paleobiological information, even knowing that in some cases the proximity to the metaphysis could limit the interpretations about the growth record and lead to underestimated skeletochronology (Stein & Sander, 2009). Before histological sampling, we measured the circumferences with a tape-measure, and the diaphysis diameter and length of the bones with a digital caliper. Because some specimens have no midshaft preserved, two circumferential measures were taken: the first at approximately the proximal 1/4 of the shaft length (common to all bones), used to standardize the comparisons within the sample; the second in the mid-diaphyseal region for the bones in which the diaphysis was best-preserved. Specimens with incomplete diaphyses were compared to complete elements, allowing the estimation of their total length. Regions with muscle scars were avoided in order to not overestimate the circumferential and diameter measures.

**Figure 2:**
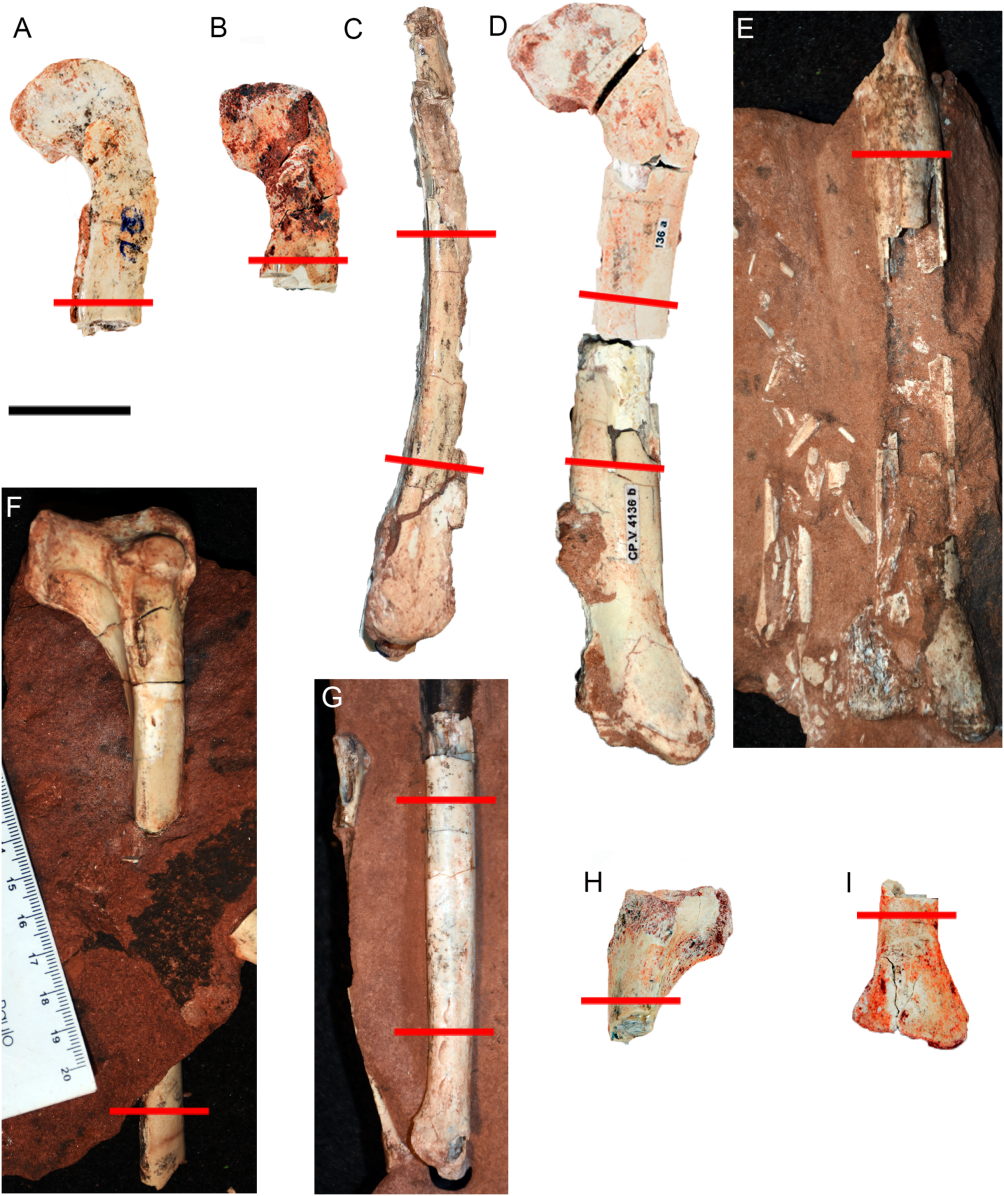
Hind limb bones of *Vespersaurus paranaensis*. (A–E) Femora. (A) CP.V 4142 (left), in anterior view. (B) CP.V 2277 (left) in anterior view. (C) CP.V 4264 (left) in lateral view. (D) CP.V 4136 (left) in anterior view. (E) CP.V 4206 (left) in posterior view. (F–J) Tibiae. (F) CP.V 4140 (left) in lateral view. (G) CP.V 4130 (right) in medial view. (H) CP.V 4312a (left) in medial view. (I) CP.V 2385 (left) in anterior view. Red lines indicate the locations where the histological cross-sections were taken. Scale bar: 3 cm.

The most incomplete bones were sectioned as close as possible to the midshaft (Fig. 2). In these elements, the sections were performed in standardized regions between the 1/4 and 1/2 of the proximal or distal length of the bone. In the near-complete specimens, the thin sections were taken at more than one level in order to access the microstructural variation along the diaphysis. The preparation of thin sections was adapted from Lamm (2007). Fossils were embedded in epoxy resin Rp031 to protect the specimens against the saw vibration. Sectioning was done by a precision router (Dremel^®^, Racine, WI, USA) after the resin hardened. Bone fragments were then soaked in epoxy resin to produce resin blocks, which were polished using a lap wheel Politriz Aropol^®^ VV200-PU and sandpapers Norton Saint-Gobain^®^ T277 with P100, P600 and P1200 lines/inch sequentially. One block surface was fixed on a histological slide using epoxy resin, while the other side was thinned and polished using the same sandpaper series.

The thin sections were numbered and deposited in the CENPALEO collection. Histological slides were analyzed using a Zeiss Axioplan Fluorescence Phase Contrast Microscope with conventional white-light reflection and transmitted circularly polarized light with 4×, 20× or 40× objectives. Images were acquired with an Axiocam ICm 1 (FireWire, 1.4MP, 1/2″, monochrome) video camera using the interface program ZEN lite^®^ software. The high-resolution whole-slide images were uploaded on the Morphobank online repository (O’Leary & Kaufman, 2012), available at the access link http://morphobank.org/permalink/?P3673.

Lines of Arrested Growth (LAGs) and annuli (Francillon-Vieillot, Arntzen & Geraudie, 1990; De Ricqlès, Castanet & Francillon-Vieillot, 2004) are formed most often in response to endogenous seasonal rhythms (Castanet & Smirina, 1990; Francillon-Vieillot et al., 1991; Castanet et al., 1993; Starck & Chinsamy, 2002), being annual indicatives (Chinsamy, 1997; Horner, Ricqlès & Padian, 2000; Erickson, 2014). The LAGs consist of cement lines that could run through the near entirety of the cross-section and indicate a complete cessation in bone growth, whereas annuli are layers of lamellar or parallel-fibered bone and denote a temporary decrease in growth rate (Francillon-Vieillot et al., 1991). Double and triple LAGs are interpreted as bi-or tri-annual growth cycles and may be counted as a single LAG representing one year in the animal’s life (Castanet et al., 1993). It is assumed that the transition from growth acceleration to deceleration, reflected by attenuated LAG spacing, may coincide with the onset of reproductive maturity (Sander, 2000; Lee & Werning, 2008). When an individual reaches somatic maturity, growth ceases and a poorly vascularized, commonly acellular, lamellar bone with a compressed pack of LAGs, the External Fundamental System (EFS), is deposited in the outermost cortex (Francillon-Vieillot, Arntzen & Geraudie, 1990; De Ricqlès, Castanet & Francillon-Vieillot, 2004; Woodward, Horner & Farlow, 2011; Lee et al., 2013).

Cortex remodeling and partial or complete hollowing of the medullary region are very common processes in bones of theropods (Erickson, 2014). This process obscures the earliest growth marks and leads to underestimated ages. For this reason, we used the theoretical “extrapolation” models proposed by Horner & Padian (2004) and modified by Lee & Werning (2008) to estimate parameter values of the inter-LAG distances and then to retro-calculate the earliest missing growth record (described below). This class of models uses the distances between the adjacent growth marks (LAGs and annuli) within the bone cortex and extrapolates to the radius of the medullary cavity (missing growth record). Before using the models to obtain a parameter of the inter-LAG space, the lost bone tissue is measured. The maximum diameters in the *X* and *Y* axis are calculated, and the intersection point is assumed as the centroid of the medullary cavity. Then, the distance between the centroid and the deepest preserved LAG is measured to obtain the missing growth space. The missing annual intervals are calculated using different theoretical models: “mean”, “maximum”, “penultimate” and “incremental factor” (Horner & Padian, 2004). In the “mean” model, the average thickness of the all preserved growth intervals is calculated and used as a parameter to reconstruct the missing LAGs. The “maximum” model assumes the thickness of the innermost preserved LAGs interval (usually the thickest) as the parameter to reconstruct the missing LAGs. The “penultimate” model assumes the thickness of the penultimate deepest LAGs interval as the parameter (in case of the innermost interval be peculiarly narrower). In the “incremental factor” model, a mean percentage of width increasing between adjacent intervals is added centripetally to the missing space. We counted the LAGs and measured the distances of the successive parts of the cross-section in each specimen. Because the results are not expected to have a normal distribution, we used the median values of the retro-calculated LAGs.

We consider these “extrapolation models” more adequate to the *Vespersaurus* sample than “overlapping models” (Lee & Werning, 2008), because the latter works better for more complete ontogenetic series, which is not the case here. “Overlapping” and “extrapolation” models are less robust in the absence of embryos and neonatal specimens. Because neonates have not been found for *Vespersaurus* so far, we assumed the hypothetical femoral and tibial mid-diaphyseal radius of neonatal femur and tibia as 1 mm (circumference of 6.3 mm and diameter of 2 mm), as calculated for *Masiakasaurus* by Lee & O’Connor (2013), because of the minimal difference in size and the close phylogenetic relationship between them. The lost portions of the growth trajectories were reconstructed considering this minimum hypothetical neonatal radius. The EFS LAGS were not used in the “extrapolation models” to retro-calculate the missing growth, but they were added to the age estimation. Age was not retro-calculated for the specimens with high incompleteness (lack of midshaft), because metaphyseal sections may lead to inaccurate numerical values.

Histological descriptions and age estimations were used to determine Femoral Histological Classes (FHC) and Tibial Histological Classes (THC). Four histological classes were established for the femora and three for the tibiae, based on bone matrix organization, bone tissue type, vascularization patterns (size and density), type and number of growth marks, spacing between growth marks, and bone remodeling degree. These data are summarized on Tables 1–3. We did not categorize femora and tibiae in the same histological classes ranking because these elements could grow at different rates in the same individual (allometry), exhibiting discrepant growth trajectories (Horner, Ricqlès & Padian, 1999; Cubo et al., 2008). Because some bones could not be sectioned in homologous regions, these categories are purely to facilitate the presentation of data. These categories do not necessarily reflect ontogeny, but some comments about this matter were made for each class.

**Table 1:** Measurements and histological features of the femora and tibiae of *Vespersarus paranaensis* that were analyzed.

Bone	Specimen	Circum ference (mm)	Growth marks	Growth Marks	Endosteal lamellae	EFS	Medulary cavity %	Bone tissue type	Ontogenetic status	Histological Class
Femur	CP.V 4136	45.86	Yes	Annuli	Yes	No	44.29%	PF	Juvenile	I
	CP.V 4142	47.90	Yes	LAGs	Yes	No	46.64 %	PF	Juvenile	II
	CP.V 4206	50.11	Yes	LAGs	Yes	No	50.36%	PF	Juvenile	II
	CP.V 4264	58.37	Yes	LAGs	Yes	No	55.63%	PF	Sexually mature	III
	CP.V 2277	*63.27	Yes	Double and triple LAGs	No (?)	No	41.50%	PF	Sexually mature	IV
Tibia	CP.V 4312a	*42.00	Yes	LAGs	Yes	No	42%	PF	Juvenile (?)	I
	CP.V 4203	–	Yes	LAGs	Yes	No	–	PF	Juvenile (?)	I
	CP.V 4140	41.29	Yes	LAGs	Yes	No	27%	PF	Juvenile/Sexually mature	II
	CP.V 4000	–	Yes	LAGs	Yes	No	–	PF	Juvenile/Sexually mature	II
	CP.V 4130	41.18	Yes	LAGs	Yes	Yes	30.78%	PF/WB	Full grown	III
	CP.V 2385	44.88	Yes	LAGs	Yes	Yes	31.78%	PF/WB	Full grown	III

**Notes:**

Circumferences in the mid-shaft in millimeters. Type of growth mark and histological features used to ontogenetic status determination.

EFS, external fundamental system; LAG, lines of arrested growth; PF, parallel-fibered bone; WB, woven bone. Asterisks represent specimens measured in the proximal diaphysis.

**Table 2:** Width of the cortical zones for femora and tibiae of *Vespersaurus paranaensis*. Zones listed from the innermost to the outermost ones. Numbers in brackets indicate intervals between LAGs within the External Fundamental System.

Bone	Specimen	Zones width average from the innermost to the outermost cortex (mm)
Femur	CP.V 4136 (midshaft) CP.V 4136 (distal half)	1.37, 0.72, 0.45 0.53, 0.48, 0.29

	CP.V 4142	0.84, 0.73, 0.64
	CP.V 4206	0.85, 0.80, 0.30
	CP.V 4264 (midshaft) CP.V 4264 (distal half)	0.69, 0.22, 0.33, 0.14 0.58, 0.23, 0.16, 0.07
	CP.V 2277	0.37, 0.15, 0.10, 0.49, 0.09, 0.41, 0.10, 0.03, 0.13, 0.01, 0.12, 0.02, 0.11, 0.05, 0.06, 0.06
Tibia	CP.V 4312a	0.24, 0.16, 0.06, 0.29
	CP.V 4203	0.59, 0.50, 0.10, 0.17
	CP.V 4140	0.79, 0.17, 0.21, 0.05, 0.04
	CP.V 4000	0.35, 0.39, 0.17, 0.11, 0.08
	CP.V 4130	0.68, 0.18, 0.24, 0.48, 0.21, 0.05, (0.01, 0.01, 0.01)
	CP.V 2385	0.61, 0.13, 0.38, 0.11, 0.23, 0.04, (0.05, 0.02, 0.01, 0.01)

**Table 3:** Growth record and age estimation of the femora and tibiae of *Vespersarus paranaensis* analyzed.

Bone	Specimen	Growth marks number	Retro-calculated LAGs ± SE (years)	Age estimation (years)
Femur	CP.V 4136	2 Annuli	3 ± 0	5
	CP.V 4142	2 LAGs	3 ± 0.62	5
	CP.V 4206	2 LAGS	–	–
	CP.V 4264	3 LAGs	5 ± 0	8
	CP.V 2277	7 LAGs (simple, double and triple)	7 ± 1.15	14
Tibia	CP.V 4312a	3 LAGs	–	–
	CP.V 4203	3 LAGs	–	–
	CP.V 4140	4 LAGs	3 ± 0.85	7
	CP.V 4000	4 LAGs	–	–
	CP.V 2385	5 LAGs (4)	4 ± 0.95	13
	CP.V 4130	5 LAGs (3)	2 ± 0.57	10

**Note:**

Number of preserved growth marks (annuli or Lines of Arrested Growth) and age estimation. LAGs within the External Fundamental System of the tibiae CP.V 2385 and CP.V 4130 are not considered. SE, standard error.

## Results

### Femoral histological classes of *Vespersaurus paranaensis*

#### Femoral histological class I (CP.V 4136)

**Figure 3:**
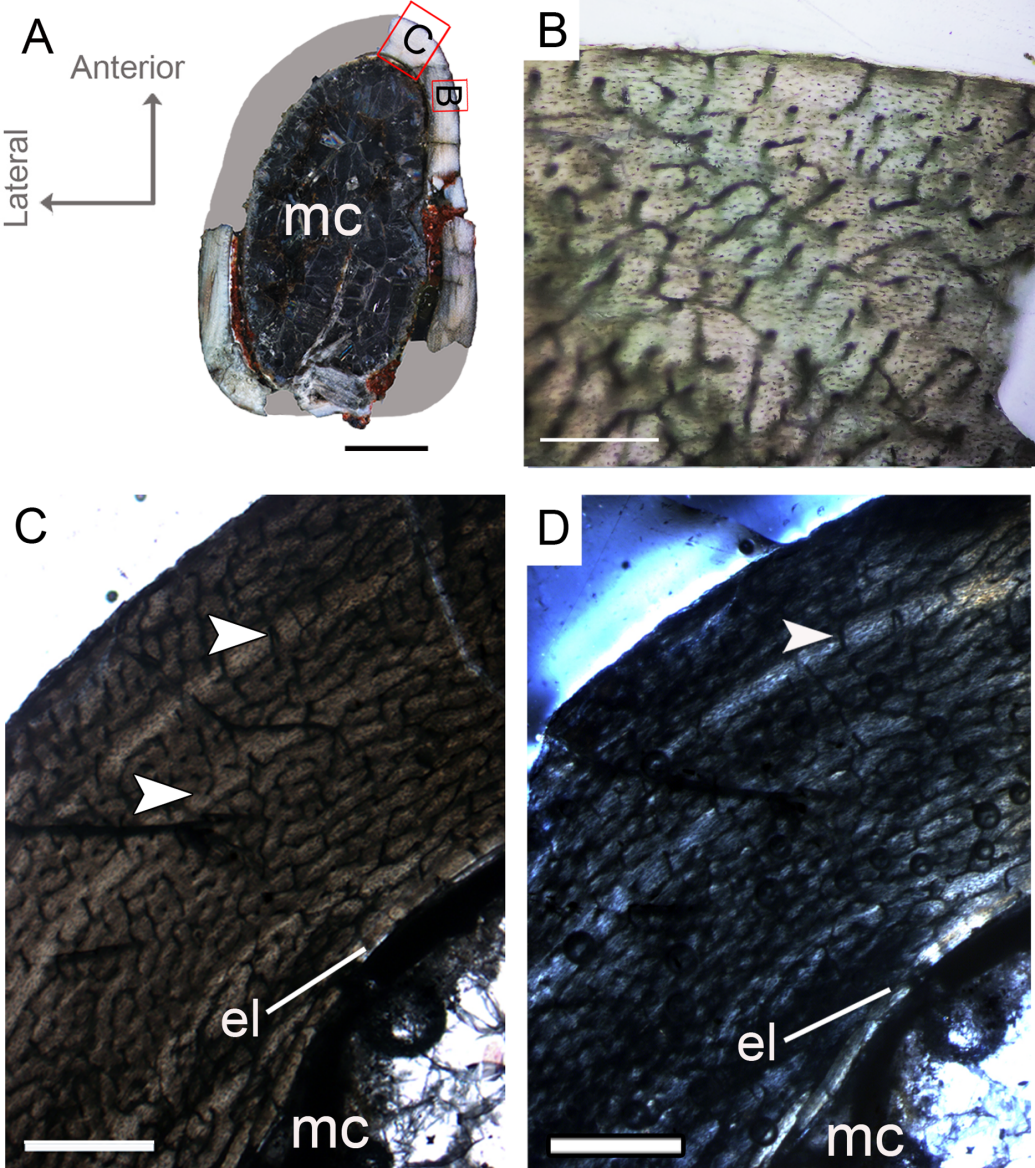
Femoral Histological Class (FHC) I of *Vespersaurus paranaensis*. (A) Whole cross-section of CP.V 4136. (B) High magnification of the deep cortex showing microstructural pattern in CP.V 4136. (C) Cortex of CP.V 4136 showing two bands of poorly vascularized lamellar bone (white arrow) interpreted as annuli. (D) The same image in polarized light. Thin layer of endosteal lamella surrounding the medullary cavity is present in this region of the cross-section, evidenced by polarized light. Abbreviations: el, endosteal lamellae; mc, medullary cavity. Scale bars: 3 mm in (A), 100 μm in (B) and 1000 μm in (C).

The complete femur CP.V 4136 is the smallest specimen of the sample. It is 182 mm in length, 16 mm in antero-posterior diameter and 45.86 mm in circumference (Table 1). We sectioned the diaphysis of this specimen at two points of the midshaft (Fig. 2D). In these sampled locations, the microstructure and the growth record did not vary along the diaphysis. It is the only specimen to have two annuli rather than any LAG (Fig. 3C; Table 2), suggesting growth with a periodic reduction in the rate of bone deposition, but without interruption. The deposition of annuli rather than well marked LAGs can occur in very young individuals (Woodward, Padian & Lee, 2013). Three cortical zones are delimited between the two annuli. The innermost zone is the thickest on average (1.37 mm), showing an initial pulse of proportionally faster growth, followed by two zones (0.72 and 0.45 mm) that decrease slightly in width outward (Table 2). The medullary cavity occupies 44.29% of the cross-sectional area, and a very tenuous endosteal lamella is visible (0.04 mm in thickness). Resorption cavities were not observed in CP.V 4136. Secondary osteons were scarce in CP.V 4136 and limited to a small area in the medial-posterior deep cortex. The cortex exhibits a high birefringence under polarized light (Fig. 3D). Osteocyte lacunae are flattened, preferentially-oriented, and arranged parallel to one another, forming a parallel-fibered matrix. Within the annuli, the osteocyte lacunae are even more flattened and preferentially-oriented. The cortex presents reticular and longitudinal channels, the later often consisting of primary osteons. Primary osteons are highly abundant in the deep cortex. The degree of vascularization (size and density of the channels) is homogeneous throughout the entire cross-section. In the subperiosteal region of this specimen, some channels are not encircled by bone matrix, such that they are open to the outside (Fig. 3B). An age retro-calculation plus the growth marks number allows us to estimate the individual should be around its fifth year of life at the time of death (Table 3). The minimal remodeling of the deep cortex, a tenuous endosteal lamella, plus the annuli number and age estimation, support a juvenile status for CP.V 4136.

#### Femoral histological class II (CP.V 4142 and CP.V 4206)

**Figure 4:**
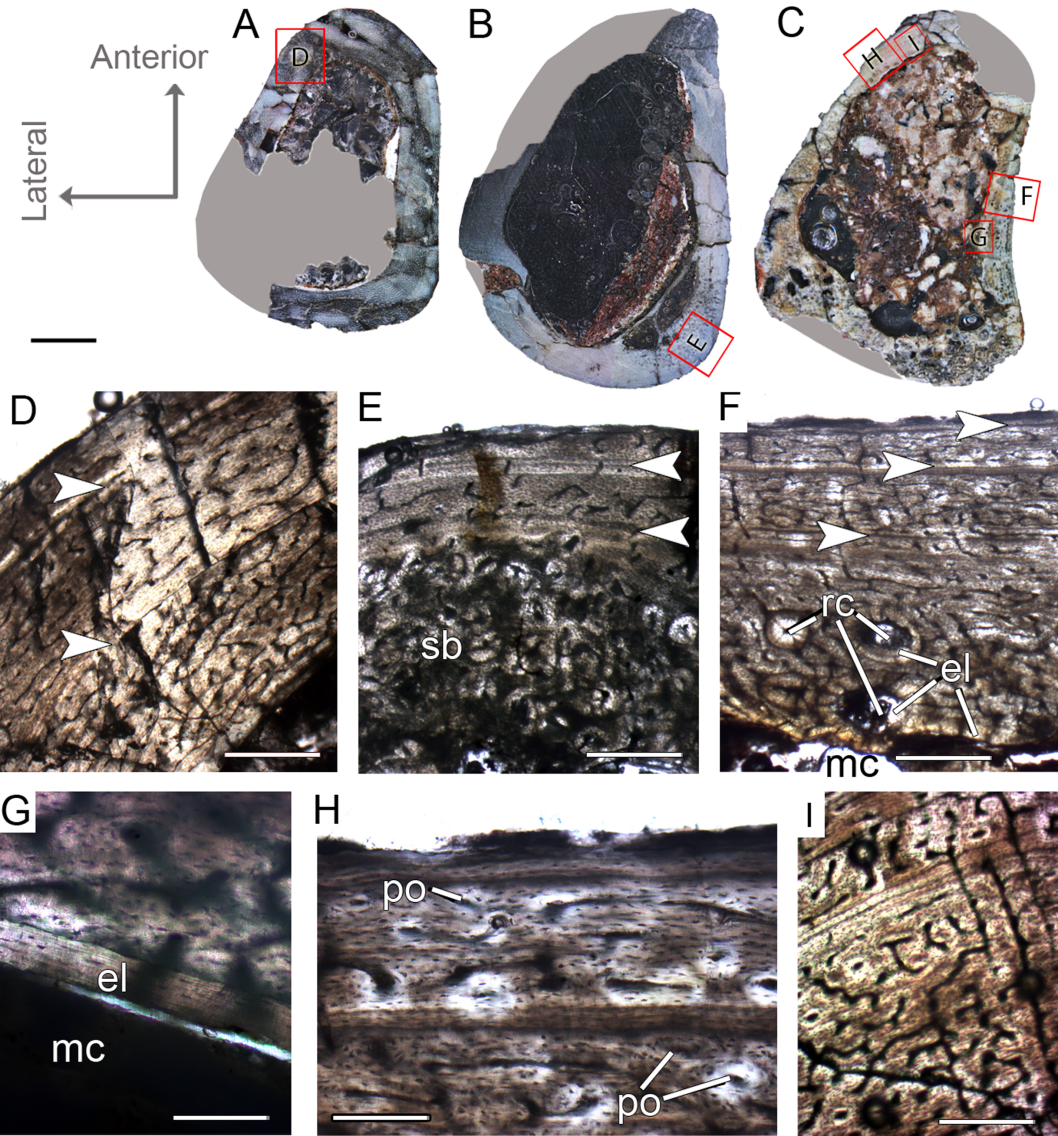
Femoral Histological Class (FHC) II and III of *Vespersaurus paranaensis*. (A–C) Whole cross-sections of femora CP.V 4142 (FHC II), CP.V 4206 (B; FHC II) and CP.V 4264 (FHC III), respectively. (D) Cortex of CP.V 4142. (E) Cortex of CP.V 4206 showing a large amount of secondary osteons and peripheral cortex poorly vascularized. (F) Cortex of CP.V4264 showing deep cortex with resorption cavities covered by endosteal lamellae. (G) High magnification of the femur CP.V 4264 showing a endosteal lamellae in detail. (H) Peripheral outer cortex showing primary osteons. (I) Cross-section of femur CP.V4264 showing double LAGs. White arrows indicate LAGs. Abbreviations: el, endosteal lamellae; mc, medullary cavity; po, primary osteon; sb, secondary bone. Scale bar: 3 mm in (A)–(C), 500 μm in (D)–(I), apart (G), which is equal to 80 μm.

The femora CP.V 4142 and CP.V 4206 are nearly equal in size, varying between 16 and 18 mm in antero-posterior diameter and 47.90–50.11 mm in diaphyseal circumference (Table 1), respectively. CP.V 4206 is severely taphonomically damaged and partially embedded in the matrix, with most of the shaft preserved as impression. The specimen conserved the proximal portion of the diaphysis (the region between the insertion of the M. iliofemoralis externus and M. caudofemoralis longus fossa) and the distal end (most of the fibular condyle). CP.V 4142 represents a proximal half of the femur shaft, with a small degree of compression. The femora CP.V 4142 and CP.V 4206 exhibit two marked LAGs, typifying FHC II (Figs. 4D and 4E). The cortical zones of the CP.V 4206 and CP.V 4142 are nearly equal in thickness, and the zone thickness decreases toward the subperiosteal cortex in both specimens (Table 2). Although CP.V 4136 (FHC I) described above also exhibits two cyclical growth marks (annuli), we prefer to discriminate CP.V 4206 and CP.V 4142 in a different class for the following reasons. In CP.V 4142 and CP.V 4206, the medullary cavities have 17.94 and 24.94 mm of circumference, which corresponds to 46.64% and 50.36% of the cross-sectional area, respectively (Table 1), a little larger than in FHC I. The medullary cavities in CP.V 4142 and CP.V 4206 are covered by thicker endosteal lamellae (0.07 mm) than seen in FHC I (0.04 mm). CP.V 4206 exhibits almost all cortical area obliterated by secondary osteons, especially in the medial-posterior zone of the cross-section, differing from CP.V 4136 (FHC I), which exhibits little secondary remodeling, at least in the sampled section. Curiously, CP.V 4142 also lacks secondary osteons, which suggests variation of secondary remodeling even in femora displaying similar size. Longitudinal and primary osteons are rare, and all vascular channels are confined within the cortex in FHC II, differing from FHC I, which exhibits a high amount of primary osteons and open vascular channels in the subperiosteal cortex. As in FHC I, the osteocyte lacunae are flattened, preferentially-oriented, and parallel one to another, forming a parallel-fibered matrix. FHC II specimens have plexiform channels in the innermost cortex, graduating to a reticular arrangement toward the outside (Fig. 4D). Age retro-calculation of CP.V 4142 allow us to estimate that the members of FHC II would be in their fifth year of life at the time of death (Table 3). Despite the same age inferred for FHC I, the set of histological features of CP.V 4142 and CP.V 4206 suggests a more mature ontogenetic status for FHC II.

#### Femoral histological class III (CP.V 4264)

The femur CP.V 4264 preserves about 80% of the shaft length (Fig. 2C). The head was not preserved, but the distal epiphysis is nearly complete. The diaphysis was severely damaged, and the anterior side of the bone was partially eroded. CP.V 4264 has an antero-posterior diameter of 14 mm and a circumference of about 58.37 mm (Table 1). Because CP.V 4264 preserved most of its diaphysis, it was sectioned in two points, at the midshaft and the distal half of the shaft (Fig. 2C). The femur CP.V 4264 is microstructurally similar to that of the FHC II, but exhibits three LAGs (Fig. 4F) instead of two.

The LAG number remains unaltered along the two sampled bone locations, and the spacing between the zones does not vary in thickness along the shaft (Table 2). The outermost LAG (3rd) is close to the outer cortex limit—0.07 mm (distal half) and 0.14 mm (mid shaft)—and seems to have been deposited near the time of the individual’s death. The medullary cavity corresponds to 55.63% of the cross-sectional area in FHC III and presents a surrounding endosteal lamella similar in thickness to that of FHC II (about 0.04 mm). Several resorption cavities are present in the perimedullary cortex of CP.V 4264, and they are significantly more abundant in the distal half of the bone shaft. The cortex consists of a parallel-fibered matrix with flattened and preferentially-oriented osteocyte lacunae. Most vascular channels are reticular, but the vascularization decreases toward the outer cortex, and simple longitudinal vascular channels become abundant at the subperiosteal zone. Primary osteons are common and more frequent in the two innermost cortical zones than the outermost one. The greater number of growth marks (three) indicates CP.V 4264 belongs to a more advanced ontogenetic stage than FHC I and II. The lesser vascularization plus the zones narrowing after the second preserved LAG suggests that CP.V 4264 was experiencing a decrease in growth typical of sexual maturity around the seventh year of life (age estimated with LAG number plus retro-calculation models) (Table 3).

#### Femoral histological class IV (CP.V 2277)

**Figure 5:**
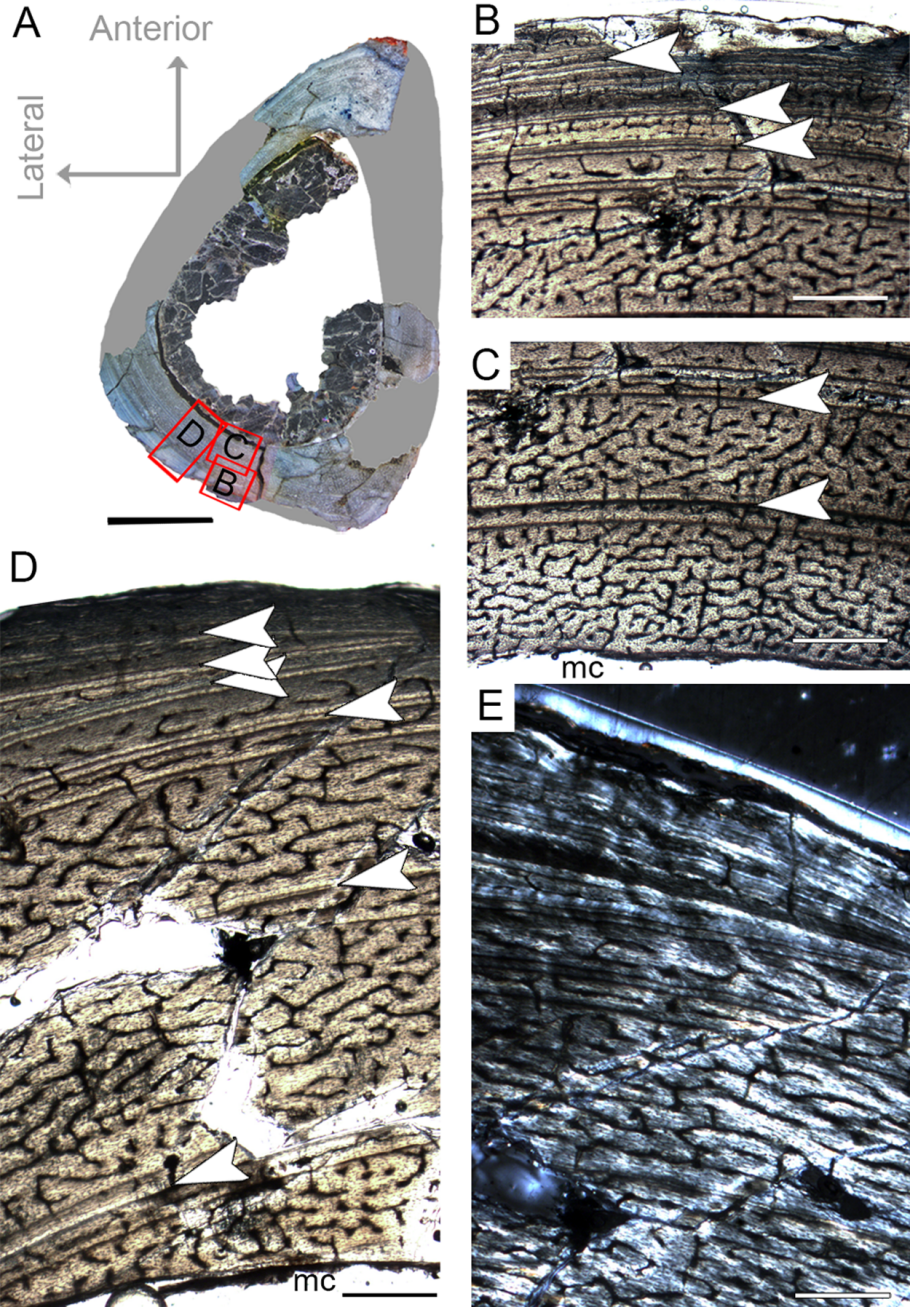
Femoral Histological Class (FHC) IV of *Vespersaurus paranaensis*. (A) Whole cross-section of CP.V 2277. (B) External and (C) internal cortex of CP.V 2277 showing the large amount of double and triple LAGs, and vascularization variation between zones (overlapping images). (D) Other cortical region of CP.V 2277 showing forked LAGs in normal and (E) polarized light. Note that specimen has no endosteal lamellae covering the medullary cavity. White arrows indicate LAGs. Abbreviation: mc, medullary cavity. Scale bar: 3 mm in (A) and 300 μm in (B)–(E).

The femur CP.V 2277 is incomplete, and the section was taken from one quarter of the proximo-distal length of the diaphysis (Fig. 2B). The specimen exhibits 63.27 mm of circumference (Table 1) and 18 mm of antero-posterior diameter. Although distant from the midshaft, the CP.V 2277 thin-section does not exhibit the cancellous bone and discontinuous LAGs typically found in the metaphyseal zone, suggesting that the section was taken in the proximal diaphysis. Despite its incompleteness, we included the femur CP.V 2277 in the sample since it was the largest femur of *Vespersaurus* found so far (Fig. 2B). In this femur, the zones of parallel-fibered bone are separated by single, double, or triple LAGs, totaling eight zones and seven packs of LAGs (assumed as bi-and tri-annual growth marks) (Fig. 5D). The innermost zone ends with a pack of double LAGs. The second zone ends with three closely spaced LAGs, forming triple LAGs. The third and fourth zones end with double LAGs each. Three single LAGs separate the last outermost zones. The zones between LAGs decrease gradually in thickness centrifugally, especially after the third zone (third pack of LAGs; Table 2). The medullary cavity has 25.9 mm of circumference and occupies 41.50% of the cross-sectional area. Curiously, endosteal lamella were not observed in CP.V 2277, which could indicate that the medullary cavity was under endosteal resorption at the time of death (Chinsamy-Turan, 2005). The secondary osteons are restricted to the deep cortex, mainly in the anteromedial, anterior and anterolateral edges of the cross-section, just near to the ridge of M. caudofemoralis longus fossa. The outer cortex is rarely occupied by primary osteons in CP.V 2277. The vascular channels are entirely reticular-type but differ both in size and number between the zones (Figs. 5B–5D). The three innermost inter-LAG zones of CP.V 2277 are nearly equal in vascularization. The fourth zone presents reticular channels like the innermost ones, but in a smaller amount. Curiously, the fifth zone has a higher concentration of channels, but they consist of smaller vascular channels. The subsequent outermost zones comprise thinner and smaller reticular channels, showing a decrease in vascularization. The subperiosteal cortex is surrounded by an avascular tissue of the lamellar bone matrix, forming a lamellar-zonal bone (Fig. 5D). However, it is important to emphasize that CP.V 2277 (FHC IV) was sectioned near the ridge where the M. caudofemoralis longus fossa is located, broadening the cortical area. This femur might be the ontogenetically oldest specimen of the sample based on the highest LAGs number, extensive secondary remodeling, and size. Nevertheless, the absence of an EFS evidences that the bone had not reached full size. Taking into account that reduced growth rate is linked to the achievement of the phase of sexual maturity in dinosaurs (Sander, 2000; Lee & Werning, 2008), and that in CP.V 2277 this is more evidenced after the third growth pulse, we can infer that, based on femora, *Vespersaurus* could reach sexual maturity between the seventh (based on femur CP.V 4264) and tenth (based on CP.V 2277) years of life.

### Tibial histological classes of *Vespersaurus paranaensis*

#### Tibial histological class I (CP.V 4203, CP.V 4312a)

**Figure 6:**
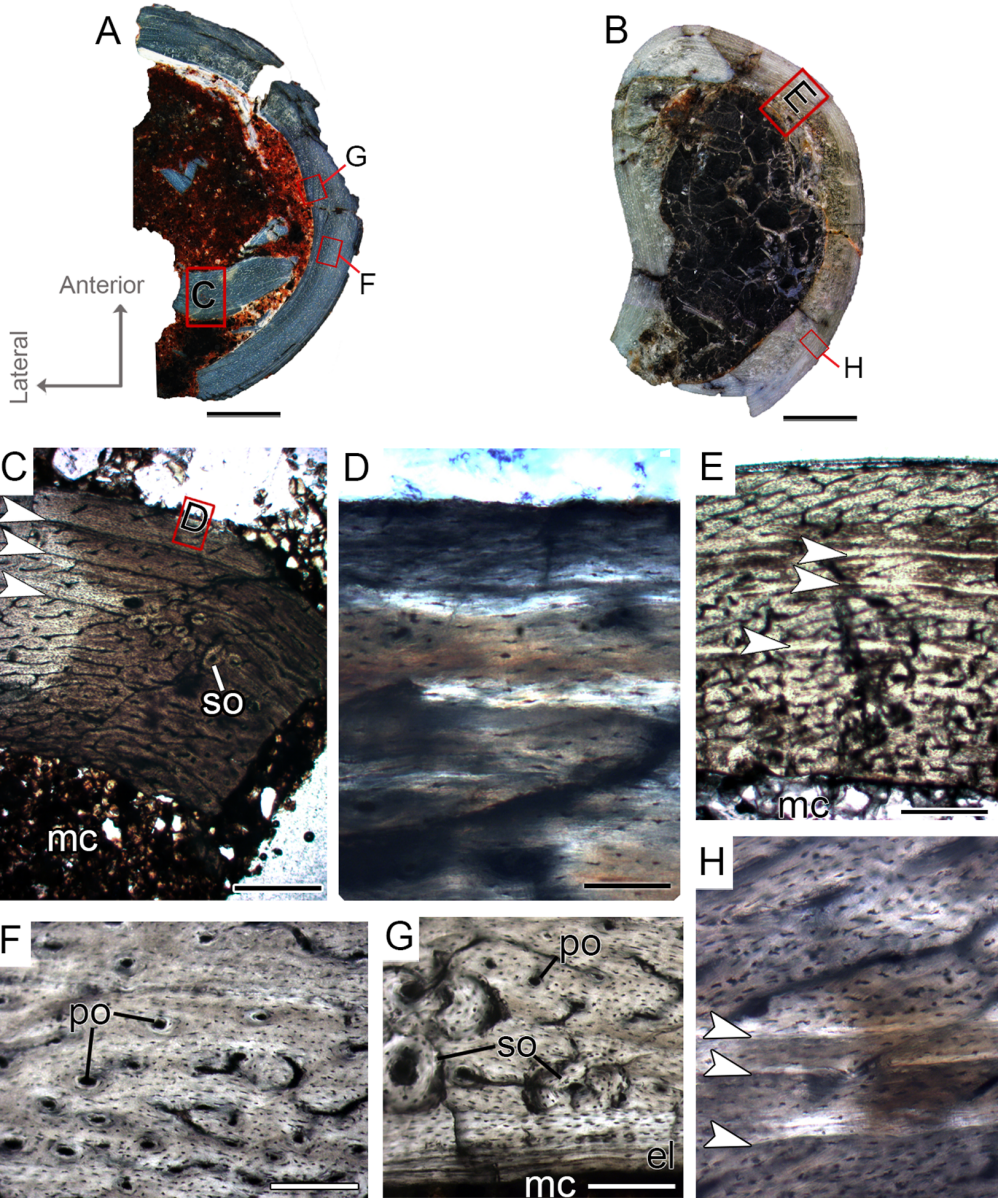
Tibial Histological Class (THC) I of *Vespersaurus paranaensis*. (A and B) Whole cross-sections of tibiae CP.V 4203 and CP.V 4312a, respectively. (C) Cortical zone detail of CP.V 4203. (D) Sub-periosteal cortex of CP.V 4203 under polarized light. (E) Cortical zone detail of CP.V 4312a. (F and G) Primary osteons and isolated secondary osteons of CP.V 4203. Note the diameter variation between vascular channels of the secondary osteons. (H) Detail of LAGs of CP.V 4312a under polarized light. White arrows indicate LAGs. Abbreviations: el, endosteal lamellae; LAG, line of arrested growth; mc, medullary cavity; po, primary osteon; sb, secondary bone, so, secondary osteon; t, trabeculae. Scale: 3 mm in (A) and (B), 300 μm in (C) and (E), 80 μm in (F) and (G) and 10 μm in (D) and (H).

CP.V 4312a comprises a fragment of the proximal end with a circumference of 42 mm (Table 1), whereas CP.V 4203 is a highly fragmented and broken proximal diaphysis that prevents circumferential measurement. CP.V 4312a and CP.V 4203 exhibit the most immature growth record among the sampled tibiae, with three LAGs each. However, caution is necessary in this matter because the growth record could be biased by the proximity of the metaphysis in CP.V 4312a (Fig. 2H). In both tibiae, the innermost zones slightly decrease in thickness toward the subperiosteal cortex, but the outermost zones are exceptionally thicker than the penultimate ones (Table 2). The bones contain relatively thin compacta surrounding the medullary cavities in THC I. Medullary cavity exhibits 21 mm of circumference, occupying about 42% of the cross-sectional area in CP.V 4312a. Due to the fragmentary state of CP.V 4306, its medullary cavity circumference was not measured. The medullary cavities are partially covered by what we interpret as possible endosteal lamellae in CP.V 4306 and CP.V 4312a (about 0.10 mm in thickness), which consists of slightly thicker layer compared to the femora of FHC I and II (0.07 mm). The deep cortex is filled extensively by secondary osteons in CP.V 4312a, whereas CP.V 4306 exhibits fewer secondary osteons. Isolated secondary osteons within the primary bone in the outer cortex are common. Both specimens exhibit high birefringence under polarized light (Fig. 5D), and preferentially oriented and flattened osteocyte lacunae characterizing a parallel-fibered bone tissue. Primary osteons are abundant in both specimens of THC I with the deep cortices showing primary and secondary osteons side by side. Plexiform vascular channels occur in the deep cortex of both THC I specimen, but in CP.V 4306 they are replaced by a less dense and reticular vascular pattern toward the subperiosteal zone suggesting that this tibia reduced vascularity after the third LAG deposition. CP.V 4312a exhibits plexiform type occupying the outermost zone with any perceptible transition due to the high amount of secondary osteons in the cortical bone. Age estimation was not applied for the specimens belonging to THC I because the zones width required for retro-calculation are obscured by the remodeling, which underestimates the results (CP.V 4312a), or because the transversal section is highly incomplete for retro-calculation (CP.V4203). Although obtaining retro-calculated age is precluded, it is possible to observe that the zone widths and degree of vascularization slightly decrease outward in the cortex after the first zone in CP.V 4203 (Fig. 6C) (not easily recognized in CP.V 4312a), which suggests that the rate of bone deposition was slowing at the time of death. However, the supposed status of sexually mature individuals for this class remains in doubt.

#### Tibial histological class II (CP.V 4000, CP.V 4140)

**Figure 7:**
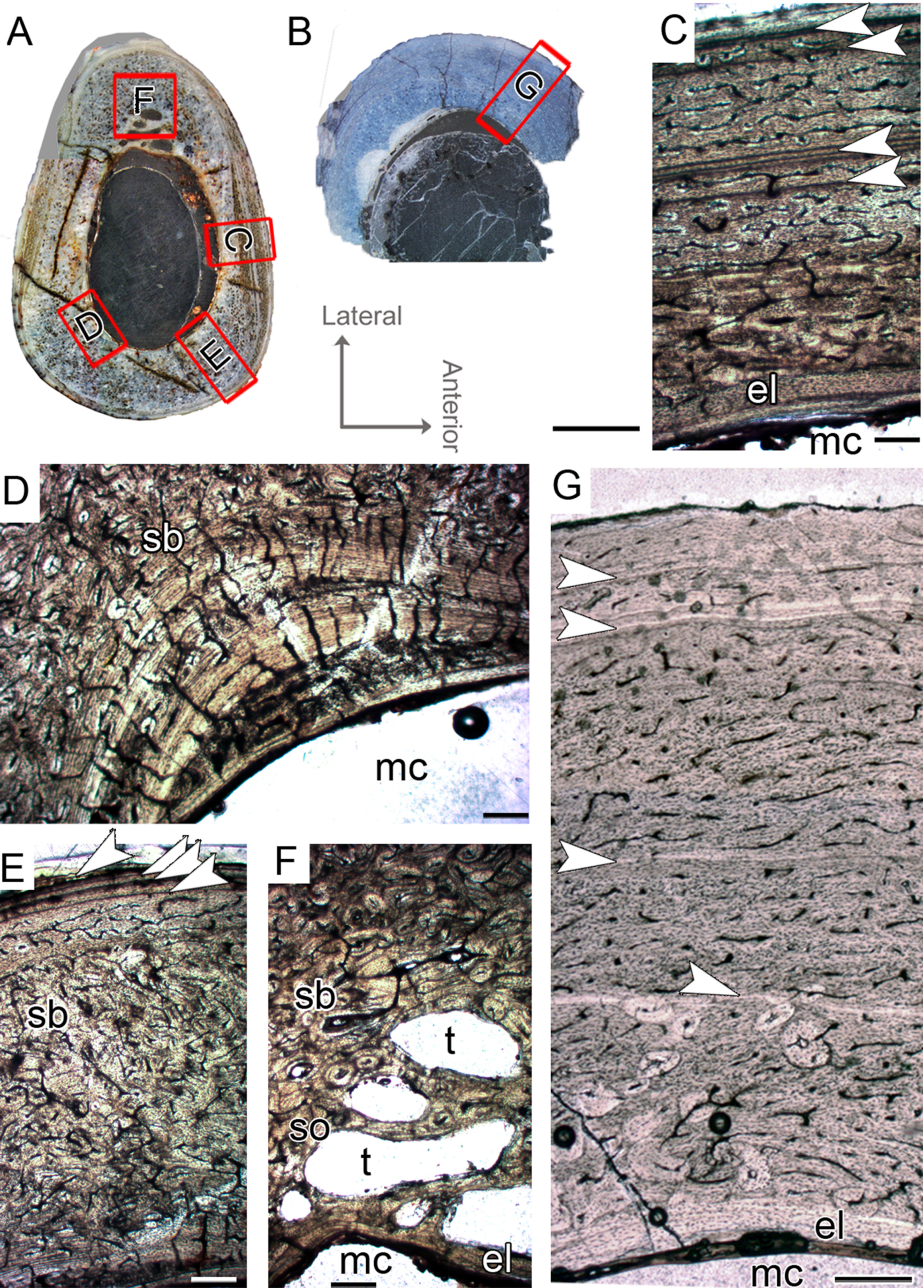
Tibial Histological Class (THC) II of *Vespersaurus paranaensis*. (A and B) Whole cross-section of tibiae CP.V 4140 and CP.V 4000, respectively. (C) High magnification of the cortex of CP.V 4140. (D) Endosteal drift in the deep cortex of CP.V 4140. (E) Large amount of Haversian (secondary) bone and close packed LAGs not forming EFS in CP.V 4140. (F) Trabecular bone with secondary osteons in perimedullary zone of the cortex in CP.V 4140. (G) High magnification of the cortex of CP.V 4000.The Haversian bone can occupy between 1/3 and 1/5 of the cortex (C and E). White arrows indicate LAGs. Abbreviations: el, endosteal lamellae; LAG, line of arrested growth; mc, in (A) and (B), 200 μm in (C), (E), (F) and (G) and 400 μm in (D). Medullary cavity; sb, secondary bone, so, secondary osteon; t, trabeculae. Scale: 3 mm.

The specimen CP.V 4000 comprises a crushed fragment of the tibial diaphysis (the circumference cannot be measured). CP.V 4140 consists of the distal half of a left tibia with a circumference of 41.29 mm (Table 1) and an antero-posterior diameter of 14 mm. Tibiae CP.V 4000 and CP.V4140 exhibit four LAGs each and they typifying the THC II. A tentative age of seven years old was inferred for CP. V 4140, but no estimation was possible for CP. V 4000 due to the incompleteness of the cross-section. A gradual decrease outward in the zones widths is observable in CP 4140 after the first LAG deposition, around the 4th year of life (Table 2), suggesting that the THC II individuals were gradually reducing their growth rate (Figs. 7C and 7G). In CP.V 4140, the medullary cavity has 12.42 mm of circumference, which corresponds to 27% of the whole cross-section (not calculated in CP.V 4000). The medullary cavity is covered by a thick layer of endosteal lamellae in these tibiae (about 0.30 mm in thickness) in both specimens, but in CP.V 4140, the endosteal lamella is expanded in the medial and lateral boundaries. In these regions (more evident on the lateral side), endosteal lamellae are filled by radial vascular channels. In the posteromedial perimedullary zone of CP.V4140, there are two layers of lamellar-zonal bone (lamellae) alternating with parallel-fibered bone (Fig. 7D). The endosteal lamellae are followed by a relatively thick cortex covering the whole medullary cavity in CP.V 4140 and CP.V 4000. Only in CP.V 4140, the anterior and posterior regions of the deep cortex exhibit trabecular bone with primary and secondary osteons (Fig. 7F). The deep cortices are extensively filled by secondary osteons, which occupy between 2/3 and 5/6 of the compacta in CP.V 4140, and between 1/2 and 2/3 in CP.V 4000, indicating intense remodeling (Fig. 7E). Osteocyte lacunae are flattened and parallel in the compacta of THC II specimens, suggesting some degree of collagen fiber orientation, which typifies the parallel-fibered matrix. For all specimens, the high birefringence under polarized light reinforces the predominance of parallel-fibered bone in the compacta. Primary osteons rarely occur in THC II, and reticular vascular channels are predominant. In CP.V 4000, there is a clear decrease in density and size of the channels toward the subperiosteal cortex, whereas in CP.V 4140 this is not evident due to the high cortical remodeling. This feature plus the narrowing of the zones toward the outermost cortex suggests the individuals of THC II were closer to attaining sexual maturity than those of THC I, but they had not reached full size at the time of death.

#### Tibial histological class III (CP.V 2385, CP.V 4130)

**Figure 8:**
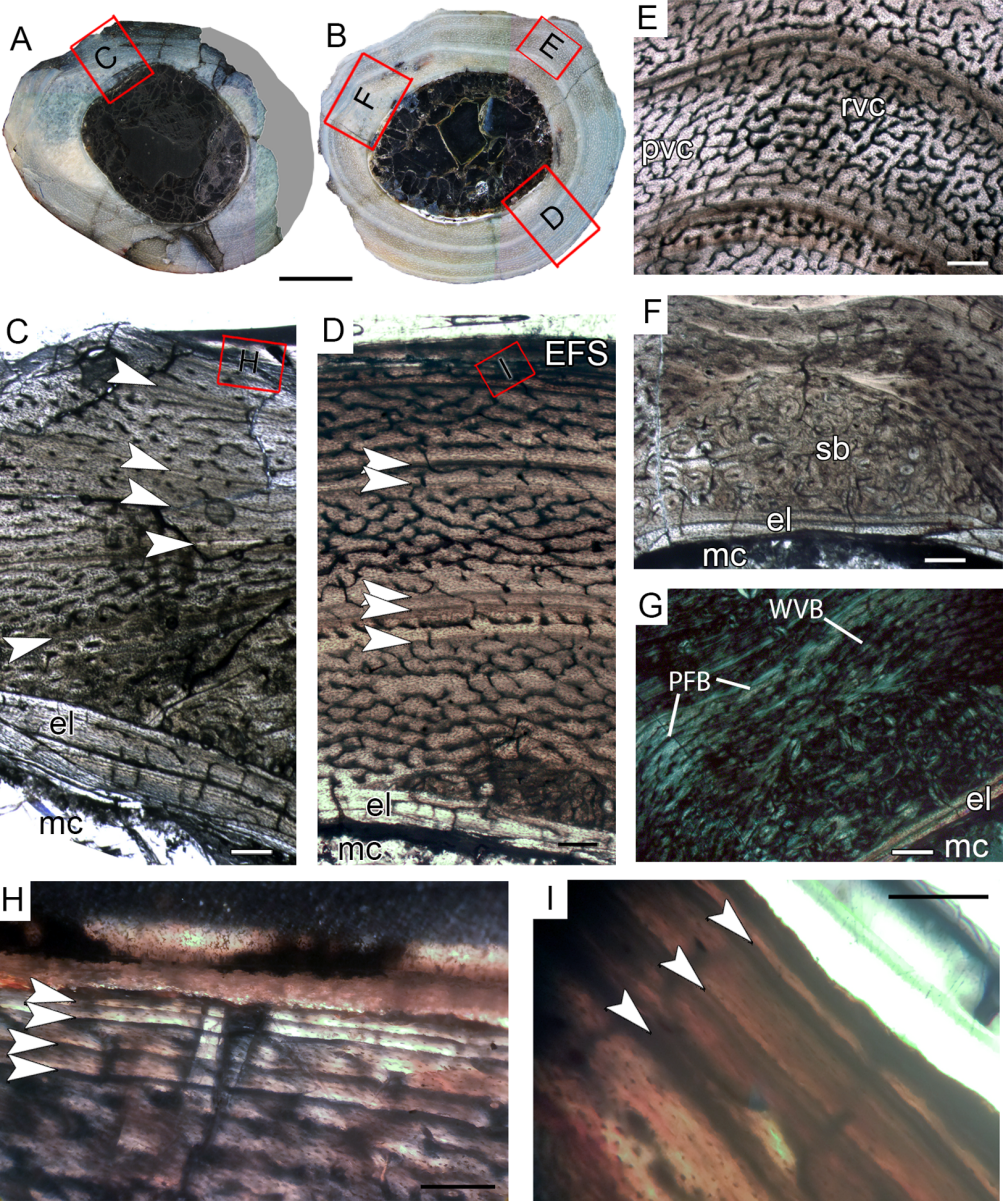
Tibial Histological Class (THC) III of *Vespersaurus paranaensis*. (A and B) Whole cross-section of tibiae CP.V 2385 and CP.V 4130, respectively. (C) Cortex of CP.V2385 showing the large and stratified endosteal lamellae. (D) Cortex of CP.V 4130 in panoramic view. (E) Variation of the vascular pattern from plexiformreticular to reticular in CP.V 4130. (F) Deep cortex of CP.V 4130 showing the bulk of secondary osteons between cortex and endosteal lamella in normal and (G) polarized light (see the small patch of woven bone). (H) Close packed LAGs within the EFS of CP.V 2385 in polarized light and (I) CP.V 4130 in normal light. White arrows indicate LAGs. Abbreviations: EFS, External fundamental system; el, endosteal lamellae; LAG, line of arrested growth; mc, medullary cavity; pb, primary bone; pvc, plexiform channels; rvc, reticular vascular channels; sb, secondary bone; wvb, woven bone. Scale bar: 3 mm in (A) and (B), 200 μm in (C)–(G), 50 μm in (H) and 100 μm in (I).

CP.V 4130 consists of atibia without the distal end, permitting sectioning at two points along the shaft (Fig. 2G). It has an antero-posterior diameter of 14 mm and a circumference of 41.18 mm (Table 1). CP.V 2385 represents a fragment of the distal end of a tibia with antero-posterior diameter of 15 mm and circumference of 44.88 mm (Table 1). This specimen does not present any sign of fusion between astragalus, calcaneus, or fibula (Fig. 2I). CP.V 2385 and CP.V 4130 both have five LAGs and are assigned to THC III. Only the members of this class have a subperiosteal cortex containing an EFS, which indicates that these individuals had completed their linear growth, at least in the tibiae (Figs. 8H and 8I). The EFS covers the entire cortex in transversal-section in both CP.V 2385 and CP.V 4130. There are four LAGs within the EFS in CP.V 2385 (totaling nine LAGs) and three in CP.V 4130 (totaling eight LAGs). Considering the retro-calculated age, we estimated 13 year of life for CP.V 2385 and 10 for CP.V 4130. Zones between LAGs decrease gradually in width toward the subperiosteal cortex in CP.V 4130 (Table 2). As described for the other cortical zones, THC III specimens exhibit a high-birefringence under polarized light (Fig. 8G). The osteocyte lacunae within the EFS are slightly more flattened than in the rest of the cortex. The EFS osteocyte lacunae are embedded within an avascular tissue of parallel-fibered matrix. The medullary cavities are surrounded by a thick compacta and are covered by thicker endosteal lamellae (about 0.40 mm in thickness) in THC III compared to the specimens of THC I and II. This could indicate the cessation of medullary cavity expansion.

The medullary cavity has 13.24 mm of circumference in CP.V 4130 (30.78% of the cross- sectional area) and 13.50 mm of circumference in CP.V 2385 (31.78% of the cross-sectional area). The deep cortex of CP.V 2385 exhibits a higher amount of secondary osteons than CP.V 4130. Primary bone consists of flattened and parallel tosteocyte lacunae. A small amount of a woven fibered matrix occurs in packs within the parallel-fibered matrix in the deep cortex, close to the secondary osteons concentration in CP.V 2385 and CP.V4130 (Fig. 8G). Along the lateral edge, the subperiosteal cortices are poorly vascularized and exhibit a layer of avascular lamellar-zonal bone. Primary osteons and simple longitudinal channels are common in the cortex of CP.V 2385, but rare in CP.V 4130. In the latter specimen, well-delimited zones of reticular and plexiform channels occur separately within the cortex. The density and size of vascular channels decrease toward the subperiosteal cortex in CP.V 2385 and CP.V 4130.

The decrease in vascularization and zone thickness outwards in the subperiosteal cortex after the first preserved LAG suggests that the specimens reached sexual maturity around three to five years of age, but they were able to grow for another four years until reaching somatic maturity, marked by the deposition of the EFS. Moreover, these specimens lived long enough to deposit three and four LAGs after entering the somatic maturity phase.

## Discussion

### Skeletal maturity in noasaurids

The assemblages of *Vespersaurus* and *Masiakasaurus* consist of immature to mature (that is, fully grown) individuals. These two noasaurines also share a similar pattern of bone matrix (parallel- fibered) and cortical growth marks as evidenced by the presence of LAGs (including double-LAGs) and annuli. In conjunction with the parallel-fibered matrix, these growth marks support slow growth rates interrupted by periodic decrease or cessation in growth from the early ontogenetic stages of *Vespersaurus* and *Masiakasaurus*.

It is stated that the achievement of the sexual maturity phase is marked by the transition to narrower cortical zones and a lesser degree of vascularization (Castanet et al., 1993; Sander, 2000; De Ricqlès, Castanet & Francillon-Vieillot, 2004; Woodward, Horner & Farlow, 2014; Lee et al., 2013). In *Vespersaurus*, the distance between LAGs towards the outer cortex reduces considerately after the innermost LAG in the tibiae CP.V 4140 (THC II) and CP.V 4130 (THC III), and after the second LAG in the femur CP.V 4264 (FHC III) and tibia CP.V 4203 (THC I). In the femur CP.V 2277 (FHC IV), the decrease in zone width occurs after the third zone, or the third pack of LAGs (Table 2). This could indicate that these individuals had already achieved sexual maturity around seven to ten years old (based on femora), or three to five years (based on tibiae). In *Vespersaurus*, the EFS is found in two tibiae of the THC III, after the fifth LAG: CP.V 2385 and CP.V 4130. This pattern is slightly different from that found in the largest tibia (UA 8685) of *Masiakasaurus*, with the EFS also placed from the sixth LAG (Lee & O’Connor, 2013), and similar to *Limusaurus*, whose fibulae exhibited EFS formation after the deposition of the fifth LAG (Wang et al., 2017). The presence of an EFS in *Vespersaurus* THC III confirms a small size even in somatically mature individuals of the Brazilian species (Langer et al., 2019). The right tibia shaft (MPCO.V 0018) assigned to the type series of *Vespersaurus* by Langer et al. (2019) has approximately the same circumference (46.18 mm) as the THC III specimens and should represent a fully grown individual, corroborating the maximum body length of 1.5 m estimated by Langer et al. (2019).

### Histovariability and functional implication

*Vespersaurus* tibiae show signs of maturity in about the third to fifth years of life, earlier than femora, which matured about seven to ten years old. No articulated femur and tibia have yet been reported for *Vespersaurus* (Langer et al., 2019), preventing evaluation of histovariability and growth record variation within the hind limb of a single individual. Therefore, we extrapolate the tibia/femur ratios of the equivalent sizes of *Limusaurus* and *Masiakasaurus* as a parameter for the Brazilian noasaurine taxon. Analyzing the available data and photos in the literature of the largest specimens (Carrano, Sampson & Forster, 2002; Xu et al., 2009; Carrano, Loewen & Sertich, 2011; Wang et al., 2017), *Limusaurus* has a tibia about 1.22 times longer than the femur, whereas this ratio is 1.10 in *Masiakasaurus*. The femora and tibiae of *Vespersaurus* with estimated length that better fit with these ratios were those of the FHC IV and THC I-II, in which tibiae were about 1.25 longer than the largest femur. This ratio is slightly higher than the tibial/femoral ratio from *Masiakasaurus* and *Limusaurus*. Based on this assumption, the femur FHC IV and the tibiae THC I-II could represent specimens from equivalent ontogenetic stages. *Vespersaurus* tibiae may complete the bone growth and deposit EFS earlier than femora, which is congruent with the different growth trajectories between femora and tibiae of *Masiakasaurus* (Lee & O’Connor, 2013). Nevertheless, we emphasize that even these data are merely an attempt to establish equivalencies between femora and tibiae of *Vespersaurus*, whereas the tibia/femur ratio may change during ontogeny.

Although we only hypothesize the equivalences between femoral and tibial classes of *Vespersaurus*, the femora and tibiae show discrepant growth records. This is expected because the growth trajectory is different in bones from the same skeleton due to the different biomechanical stresses, injury, illness, feeding or other individual particularities (Padian, Werning & Horner, 2016). Additionally, inherent bone growth processes can generate variations in the growth record. Metaphyseal regions, which undergo remodeling and extensive cortical drift, show a truncated growth record when compared to midshaft diaphyseal regions. Also, the aforementioned factors may also result a different growth record in bones of equal or similar size, as observed between FHC I and II, or between THC III specimens. Thus, different growth records may be obtained from the same skeleton and even the same bone (Horner, Ricqlès & Padian, 1999; Erickson & Tumanova, 2000; Horner, Ricqlès & Padian, 2000; Chinsamy-Turan, 2005; Woodward & Lehman, 2007; Bourdon et al., 2009).

Heterogeneous maturity among *Vespersaurus* hind limb bones could result from differential evolutionary allometry scaling. In abelisauroids, the length of the more proximal elements such as the ilium (*k* = 1.24) and femur (*k* = 1.17) scale more positively to the body length, decreasing toward the more distal elements, and being isometric in tibiae (*k* = 1.00) and metatarsals (*k* = 0.96) (Grillo & Delcourt, 2017). In addition, the growth profiles of *Masiakasaurus* showed that femora grow circumferentially more slowly than tibiae, completing full growth one year later than tibiae (Lee & O’Connor, 2013).

As a final note, the fusion between the distal end of the tibia and proximal tarsals is recognized as an indication of maturity for many non-tetanuran theropods (Tykoski, 2005) and therefore regarded as an ontogeny-dependent character in some phylogenetic matrixes (Rowe & Gauthier, 1990; Carrano, Sampson & Forster, 2002; Carrano & Sampson, 2008). However, the fusion of these bones forming a tibiotarsus has also been considered as a synapomorphy for Ceratosauria (Gauthier, 1986; Rowe, 1989; Rauhut, 2003). Other taxa occasionally fuse their fibula with the ascending astragalus process (Carrano & Sampson, 2008), or even the astragalus and calcaneus to each other but not with the tibia (Bonaparte, 1991). *Limusaurus* displays this last condition in the adult but also fuses the astragalus-calcaneus complex to the tibia in larger specimens. The distal portions of the tibia and fibula fuse to the astragalus and calcaneus in several *Masiakasaurus* specimens. In short, such co-ossifications are widely distributed and quite variable among early neotheropods (Griffin, 2018). The biological meaning of these fusions is not clear, but they confer some degree of immobility to the foot (Carrano, Loewen & Sertich, 2011).

Tibiotarsal fusion in coelophysoids is the last ontogenetic transformation to happen (Tykoski, 2005; Griffin, 2018), but this phenomenon was not evaluated for Abelisauroidea. The tibiae THC III of *Vespersaurus* represent fully-grown individuals (with EFS) and does not show any signs of being fuzed with the proximal tarsal elements. Therefore, these bones might not fuse in *Vespersaurus*, or may fuse at a very late ontogenetic stage.

### Size, growth rate and paleoecology implications

Femora and tibiae of *Vespersaurus* and *Masiakasaurus* differ microstructurally from most other Mesozoic dinosaurs taxa by the presence of a predominantly parallel-fibered bone throughout the cortex rather than the typical fibrolamellar bone complex (Horner, Ricqlès & Padian, 2000; Padian, Horner & Ricqlès, 2004; Bybee, Lee & Lamm, 2006). Whereas the latter bone tissue exhibits faster growth rates, parallel-fibered and lamellar bone are characteristic of slower growth rates (Ricqlès, 1980; Castanet et al., 2000; De Margerie, Cubo & Castanet, 2002). Despite the fibrolamellar bone complex being plesiomorphic for Ornithodira and widespread throughout the entire Dinosauria group (Ricqlès, 1980; Padian, Horner & Ricqlès, 2004; Chinsamy-Turan, 2005), there are some exceptions. Deviation from parallel-fibered bone in some small dinosaurs (total adult lengths of 1.5 m or less) was interpreted as a secondary acquisition (Padian, Horner & Ricqlès, 2004). This is the case of some small basal thyreophorans such as *Scutellosaurus* and the “hypsilophodontian” *Orodromeu*s that present parallel-fibered bone in their cortices (Padian, Horner & Ricqlès, 2004). This kind of bone tissue was also found in *Laquintasaura* and *Fruitadens*, other two small (ca. 1 m body length) basal ornithischians, showing the lack of high bone growth rates even in early ontogeny (Barrett et al., 2014). The smallest specimen of a growth series of unidentified “hypsilophodontians” from Australia displayed well-vascularized woven bone without any LAG typical of fast growth, but the larger femora (between 18 and 30 cm length) exhibit parallel-fibered tissue with LAGs in the outer cortex, testifying that growth slowed significantly through ontogeny (Woodward et al., 2011). In a few small theropod taxa, the parallel-fibered tissue occurs in alternation with the fibrolamellar bone throughout the cortex (Erickson et al., 2009; Lee & O’Connor, 2013). The long bones (e.g., femora) of *Archaeopteryx* are composed of nearly avascular parallel-fibered tissue, a pattern also observed in the early Cretaceous basal birds *Sapheornis* and *Jeholornis* (Erickson et al., 2009). This femoral histology is size-related, forming a scale-dependent continuum in which, from larger to smaller Deinonychosauria, there was a simplification in vascular complexity and better organization of fiber orientation (woven to parallel-fibered). The smallest deinonychosaur, *Mahakala* (dromaeosaurid), with a femoral length of 76 mm, shows solely parallel-fibered bone-like *Archaeopteryx* and the other cited basalmost birds (Erickson et al., 2009).

Except for the Paravian clade, the case of the noasaurines *Vespersaurus* and *Masiakasaurus* (Lee & O’Connor, 2013) seems to be one of the only among Mesozoic non-avian theropods, because the parallel-fibered bone occurs throughout the cortex in all femora and tibiae analyzed. Within Abelisauroidea, the predominance of parallel-fibered bone of these noasaurines contrasts with the fibrolamellar bone observed in the large abelisaurids *Aucasaurus* (Cerda, 2015) and *Quilmesaurus* (Baiano & Cerda, 2017). Conversely, the well-oriented collagen fibers in the abelisaurid MMCh-PV 69 femur features parallel-fibered bone (Canale et al., 2016). This similarity between the noasaurines and MMCh-PV 69 (one of the smallest abelisaurids) reinforces that a smaller body size is positively related to the slowly formed parallel-fibered bone within Abelisauroidea (Cerda, 2015; Baiano & Cerda, 2017), a trend also observed in other small dinosaur taxa (Padian, Horner & Ricqlès, 2004; Erickson et al., 2009; Woodward et al., 2011). Nevertheless, concerning Noasauridae the paleohistological pattern of *Vespersaurus* and *Masiakasauru*s shows similarity with that of the unnamed specimen CPPLIP 1490 from Late Cretaceous of Brazil (Martinelli et al., 2019), but does not with that of the Chinese elaphrosaurine *Limusaurus*. It is important to note that in the study performed by Wang et al. (2017) that the histological description of the fibulae of *Limusaurus* is only presented in the Supplemental Information and the fibrolamellar tissue is mentioned in only two of the five analyzed elements. *Masiakasaurus*, in turn, was well-sampled (four femora and three tibiae), and there is a predominance of parallel-fibered bone in all of the seven size classes (Lee & O’Connor, 2013). Lee & O’Connor (2013) mention (without illustration) that only one femur and one tibia (with EFS), from the largest specimen of the sample, revealed a very small amount of fibrolamellar bone in the caudomedial cortex and woven bone in the inner cortex, respectively. Interestingly, the THC III specimens of *Vespersaurus* also revealed small packs of woven fibered matrix within the parallel-fibered deep cortex.

Lee & O’Connor (2013) suggested the deviation from the standard dinosaurian growth pattern in *Masiakasaurus*, with the predominance of parallel-fibered bone despite fibrolamellar complex, could represent a heterochronic process such as neoteny or progenesis. The authors inferred that the growth was about 40% slower in *Masiakasaurus* than in other non-avian theropods of comparable size (e.g., *Conchoraptor*, *Byronosaurus*, *Coelophysis*), characterized by the presence of fast-growing fibrolamellar bone. This deviation would be advantageous for reducing the maintenance costs in poor resource habitats characterized by a seasonally dry climate such as the Maeverano Formation in Late Cretaceous where *Masiakasaurus* came from (Rogers et al., 2007; Lee & O’Connor, 2013). This could also be true for *Vespersaurus*, because the Caiuá Group is interpreted as a semiarid environment, like the Maeverano Formation (Rogers et al., 2007). An arid and hot climate is also interpreted for the Adamantina Formation (Goldberg & Garcia, 2000) where CPPLIP 1490 (Martinelli et al., 2019) was recovered. These shared environmental conditions experienced by the aforementioned noasaurids in the Cretaceous of Gondwana may reinforce the hypothesis of Lee & O’Connor (2013) that environmental constraints played a role in selecting slower growth rates in response to restrictive conditions of water and food supply, for example. In counterpoint to this, Woodward et al. (2011) concluded that the small Cretaceous “hypsolophodontians” from the extreme polar climate of Australian high latitudes (cold desert) exhibit patterns of growth dynamics and physiology that match with those observed in dinosaurs from lower latitudes. They considered that the inhospitable climate would not have been a driver for a slower type of growth in these animals (represented by the parallel-fibered matrix), but an exaptation that would provide a saving of energy in the face of an extreme and hostile environment.

Although further studies on this topic are still needed, we are inclined to advocate that, as in other small dinosaurs, the prevalent parallel-fibered matrix of *Vespersaurus* and *Masiakasaurus* (plus CPPLIP 1490) long bones was secondarily acquired and mainly correlated to small size but linked to ecologic conditions (arid environment) that also must have favored slow growth. Further paleohistological studies of Abelisauroidea and the investigation of bone tissue patterns in other noasaurids (e.g., *Elaphrosaurus*, *Ligabueino*), the largest noasaurine *Austrocheirus isasii* (Ezcurra, Agnolin & Novas, 2010), or the smallest abelisaurid *Genusaurus sisterornis* (Accarie et al., 1995) could provide further test if parallel-fibered bone might be synapomorphic to Noasaurinae.

## Conclusions

This histological study described the microstructural pattern of bone tissue observed in femora and tibiae of the *Vespersaurus* assemblage in a comparative context. The peculiar predominance of parallel-fibered bone in the noasaurines *Vespersaurus* and *Masiakasaurus* throughout all size classes reflects a uniformly slow growth rate even during earlier ontogenetic stages. This pattern of growth, unusual in Mesozoic dinosaurs, was secondarily acquired and is correlated to the small size of these taxa alongside life in a hostile (arid) environment. Although further studies on the osteohistological patterns of Abelisauroidea are still needed, we hypothesize that this bone pattern tissue represents a potential apomorphy for Noasaurinae.

The assemblage of *Vespersaurus* represents different “histological classes” and different ontogenetic stages. Three to four histological classes according to the sampled bone element (femora or tibiae) were established, encompassing juveniles (hypothesized to be about five years in age), sexually mature individuals, and full growth adults (with EFS). As seen in *Masiakasaurus*, *Vespersaurus* tibiae seem to deposit the EFS earlier than femora. This study confirms the small size of 1.5 m body length estimated for somatically mature individuals of *Vespersaurus paranaensis*.

## Institutional Abbreviations

CP.V: Vertebrate Paleontological Collection of Centro Paleontológico (CENPALEO) of Universidade do Contestado, Mafra, Santa Catarina, Brazil
CPPLIP: Centro de Pesquisas Paleontológicas L. I. Price of Universidade Federal do Triângulo Mineiro
UFTM: Peirópolis, Uberaba, Minas Gerais, Brazil
FMNH: Field Museum of Natural History, Chicago, IL, USA
MMCh: Museo Municipal Ernesto Bachmann, Villa El Chocón, Neuquén, Argentina
UA: Université d’Antananarivo, Antananarivo, Madagascar

## Additional Information and Declarations

### Data Availability

The following information was supplied regarding data availability: All the data used can be found in Tables 1–3. The high-resolution whole-slide images are available at Morphobank online repository: Project 3673: G. A. De Souza, M. B. Soares, A. S. Brum, M. Zucolotto, J. M. Sayão, L. C. Weinschütz, A. W. A. Kellner. 2020. Growth dynamics and osteohistology of the Brazilian noasaurid Vespersaurus paranaensis Langer et al., 2019 (Theropoda: Abelisauroidea). PeerJ. (In Press) DOI 10.7934/P3673 All the sampled specimens are housed at CENPALEO—Universidade do Contestado, Mafra, Brazil, acession numbers femur CP.V4136; femur CP.V4142; femur CP.V4206; femur CP.V4264; femur CP.V2277; tibia CP.V4312a; tibia CP.V4203; tibia CP.V4140; tibia CP.V4000; tibia CP.V4130; tibia CP.V2385.
